# Active and sham transcranial direct-current stimulation (tDCS) plus core stability on the knee kinematic and performance of the lower limb of the soccer players with dynamic knee valgus; two armed randomized clinical trial

**DOI:** 10.3934/Neuroscience.2025017

**Published:** 2025-07-21

**Authors:** Hadi Mohammadi Nia Samakosh, Maedeh Maktoubian, Seyyed Pedram Rouhani Doost, Rafael Oliveira, Georgian Badicu, Sameer Badri Al-Mhanna, Mahdieh Hassanzadeh, Peyman Amadekhiar, Reza Rezaeian Vaskasi

**Affiliations:** 1 Department of Biomechanics and Corrective Exercises and Sports Injuries, University of Kharazmi, Tehran 15719–14911, Iran; 2 Department of Sport Sciences and Health, Faculty of Sport Sciences and Health, University of Tehran 1417935840, Iran; 3 Department of Sports Physiology, Faculty of Physical Education and Sports Sciences, University of Mazandran 1353447416, Babolsar, Iran; 4 Santarém Polytechnic University, School of Sport, Rio Maior 2040–413, Portugal & Research Center in Sport Sciences, Health Sciences and Human Development (CIDESD), Santarém Polytechnic University, Rio Maior 2040–413, Portugal; 5 Department of Physical Education and Special Motricity, Faculty of Physical Education and Mountain Sports, Transilvania University of Braşov, Braşov, 500068, Romania; 6 Center for Global Health Research, Saveetha Medical College and Hospitals, Saveetha Institute of Medical and Technical Sciences, University of Saveetha, Chennai 602105, India; 7 Department of Elementary Education, Faculty of Hazrat Fatemeh Al-Zahra, Farhangian University of Ghaemshahr 1998963341, Mazandaran, Iran; 8 Department of Corrective Exercises and Sports Injuries, Faculty of Sport Sciences, University of Shomal 4616184596, Mazandaran, Iran

**Keywords:** dynamic knee valgus, transcranial direct-current stimulation, core stability, knee kinematics, soccer, injury prevention

## Abstract

Dynamic knee valgus (DKV) is a prevalent risk factor for anterior cruciate ligament (ACL) injuries in soccer players, particularly during noncontact mechanisms. Transcranial direct-current stimulation (tDCS) and core stability exercises have shown promise in enhancing motor control and biomechanical alignment, but their combined effects on DKV remain unexplored. This study aimed to evaluate the efficacy of active versus sham tDCS combined with core stability exercises on knee kinematic alignment and lower limb performance in young male soccer players with DKV. In this double-blind, randomized controlled trial, 42 male soccer players (aged 18–25 years) with DKV were randomly assigned to either an active tDCS group (n = 21) or a sham tDCS group (n = 21). Both groups underwent 8 weeks of core stability exercises (3 sessions/week, 30 minutes/session) preceded by 15 minutes of active (2 mA) or sham tDCS targeting the primary motor cortex (M1). The primary outcome was the frontal plane projection angle (FPPA) during a single-leg landing task, measured using 2D video analysis. Secondary outcomes included vertical jump height and 8-hop test time. Outcomes were assessed at baseline and post-intervention. A 2 × 2 mixed-model ANOVA with Bonferroni-corrected post hoc tests was used for statistical analysis via SPSS_27_. The active tDCS group showed significantly greater improvements in FPPA (+5.65% vs. +2.26%, p < 0.001, *η*p² = 0.82), vertical jump height (+25.30% vs. +10.45%, p < 0.001, *η*p² = 0.75), and 8-hop test time (−21.05% vs. −14.27%, p < 0.001, *η*p² = 0.68) compared to the sham group. Both groups improved from baseline, but the active tDCS group exhibited larger effect sizes across all outcomes. Active tDCS combined with core stability exercises significantly enhanced knee kinematic alignment and lower limb performance in soccer players with DKV compared to sham tDCS. These findings suggest that neuromodulation, when paired with targeted exercise, is a promising strategy for injury prevention and performance enhancement in athletes. Further research is needed to explore long-term effects and applicability to diverse populations.

## 1. Introduction

Soccer, one of the most popular sports globally and in Iran [1] s associated with a high risk of injuries, particularly anterior cruciate ligament (ACL) injuries, which are among the most prevalent in the sport [2]. ACL injuries are especially common in young, novice soccer players compared to their adult counterparts [3]. A 10-year survey (2005–2015) of children aged 5–14 years reported 320 ACL injuries, with 96.9% occurring in the 10–14 age group, and sports activities accounting for 56.6% of these injuries [4]. In the general population, the annual incidence of ACL injuries is 86.6 per 100,000 people, rising significantly among professional (150–370 per 100,000) and amateur athletes (30–162 per 100,000) [5],[6]. Most ACL injuries in sports like soccer, basketball, and handball occur through noncontact mechanisms, often linked to dynamic knee valgus (DKV) during landing, turning, or deceleration maneuvers [7]. DKV is fundamentally a problem of neuromuscular control, characterized by medial knee collapse due to suboptimal muscle activation patterns, including weakness or delayed activation of hip abductors and external rotators (e.g., gluteus medius and maximus) and overactivity of adductors and internal rotators [8],[9]. This misalignment results in hip adduction, internal rotation, tibial abduction, and increased knee stress, heightening the risk of ACL injury [7]. Two-dimensional video analyses have quantified DKV in the sagittal plane, confirming its role as a risk factor for lower limb injuries, particularly in sports requiring frequent jumping and landing [10]–[12]. DKV also contributes to acute injuries, such noncontact ACL tears, and chronic conditions, such as patellofemoral pain [13],[14]. Video and cadaveric studies have identified DKV, often accompanied by tibial internal rotation, as a primary mechanism of ACL injury [15]–[18]. The iliotibial band, influenced by the tensor fascia lata and gluteus maximus, stabilizes the knee in the frontal plane but can exacerbate tibial abduction and valgus under excessive tension [19],[20]. Furthermore, in a study under non-weight-bearing conditions, it was demonstrated that the greater the load on the iliotibial band, the greater the anterior translation of the tibia and the valgus rotation of the tibia, which may affect the valgus dynamics of the knee [21]. In addition, mechanoreceptors located in tendons (Golgi tendon organ) and in muscles (muscle spindles) that are responsible for the reflex function, help the positioning of organs [22]. Disturbances of these mechanisms, which are the deep feedback system, may contribute to ACL injuries. DKV also increases patellofemoral joint stress, leading to pain and dysfunction [23].

Correcting inappropriate movement patterns may help prevent ACL injuries and other lower extremity injuries, both of which have modifiable risk factors [24]. People with poor movement quality benefit most from exercise programs, and researchers have worked to develop efficient exercise programs and advanced techniques to enhance it [25]. The effectiveness of core stability exercises as one of the means of increasing physical and movement fitness has been the subject of some current studies in this field [26],[27]. Core stability exercises not only enhance local muscle strength (“hardware”) but also re-educate motor programs at a central level (“software”), improving pelvic and trunk stability, which influences lower limb alignment via central nervous system (CNS) reprogramming [28]. It seems that plyometric training programs with emphasis on knee alignment and optimal landing methods during intense activities reduce knee valgus angle and ground reaction forces [29]. Furthermore, by using rapid forces, such training increases feedback and feedforward activities while adapting muscle and joint receptors [26]. Core stability exercises will aim to reduce DKV of the knee and improve sports performance by correcting knee posture, two variables that have a significant impact on injury prevention. Furthermore, it is unclear to what extent the various components of such programs contribute to reducing the risk of injury [30]. It significantly reduces peak landing forces, knee valgus and varus moment and improves single-limb stability, balance, knee valgus angle and landing error score [30].

Innovative approaches, such as transcranial direct-current stimulation (tDCS), may further enhance movement correction and athletic performance. tDCS is a noninvasive brain stimulation technique that modulates cortical excitability using low-intensity electrical currents (1–2 mA) applied for 20–30 minutes [31]. Anodal tDCS over the primary motor cortex (M1) increases cortical excitability, creating a window of opportunity for neuroplasticity, which facilitates the consolidation of correct motor patterns learned during exercises [32]. Active tDCS targets specific brain regions, such as the primary M1, to enhance neuronal activity, while sham tDCS mimics the procedure without delivering therapeutic current, controlling for placebo effects [31],[33]. The combination of tDCS and core stability exercises is hypothesized to enhance motor learning and neuromuscular control through complementary mechanisms. Anodal tDCS increases excitability in the primary M1, enhancing neural drive to muscles critical for knee alignment, such as the gluteus medius and vastus medialis, while core stability exercises strengthen these muscles and retrain movement patterns. This synergy may amplify neuroplastic changes in motor control networks, improving the efficiency of feedback and feedforward mechanisms, which are essential for maintaining proper knee kinematics during dynamic movements. Specifically, tDCS may enhance the CNS's ability to consolidate motor patterns practiced during core stability training, leading to more rapid and sustained reductions in DKV by optimizing muscle activation timing and coordination [18],[32]. Recent studies have shown that tDCS enhances lower limb motor control and muscle activation. For instance, Moshashaei et al. (2024) found that anodal tDCS improved feedforward activity of lower extremity muscles in female taekwondo athletes with DKV, suggesting enhanced neural drive to muscles like the gluteus medius and vastus medialis, which are critical for controlling knee valgus [18], while Sánchez-Barbadora et al. (2025) reported enhanced lower limb performance with tDCS-augmented training [22]. Jeong et al. (2021) demonstrated that core strength training alters neuromuscular risk factors for ACL injuries by improving hip and trunk stability [34]. However, no prior studies have investigated the combined effects of tDCS and core stability exercises on DKV in male soccer players, representing a critical research gap.

This study aims to evaluate the effects of active versus sham tDCS combined with core stability exercises on knee kinematic alignment (frontal plane projection angle [FPPA]) and lower limb performance (vertical jump and 8-hop test) in young male soccer players with DKV. We hypothesized that tDCS enhances exercise-induced motor learning, resulting in rapid and effective DKV correction when combined with core stability.

## 2. Materials and methods

### 2.1. Study design

This study is two-armed randomized controlled trial (RCT) with primary (kinematic) and secondary (performance) outcomes and double-blind trials, and participants and researchers are unaware of group assignments.

### 2.2. Ethics approval of research

**Figure 1.**
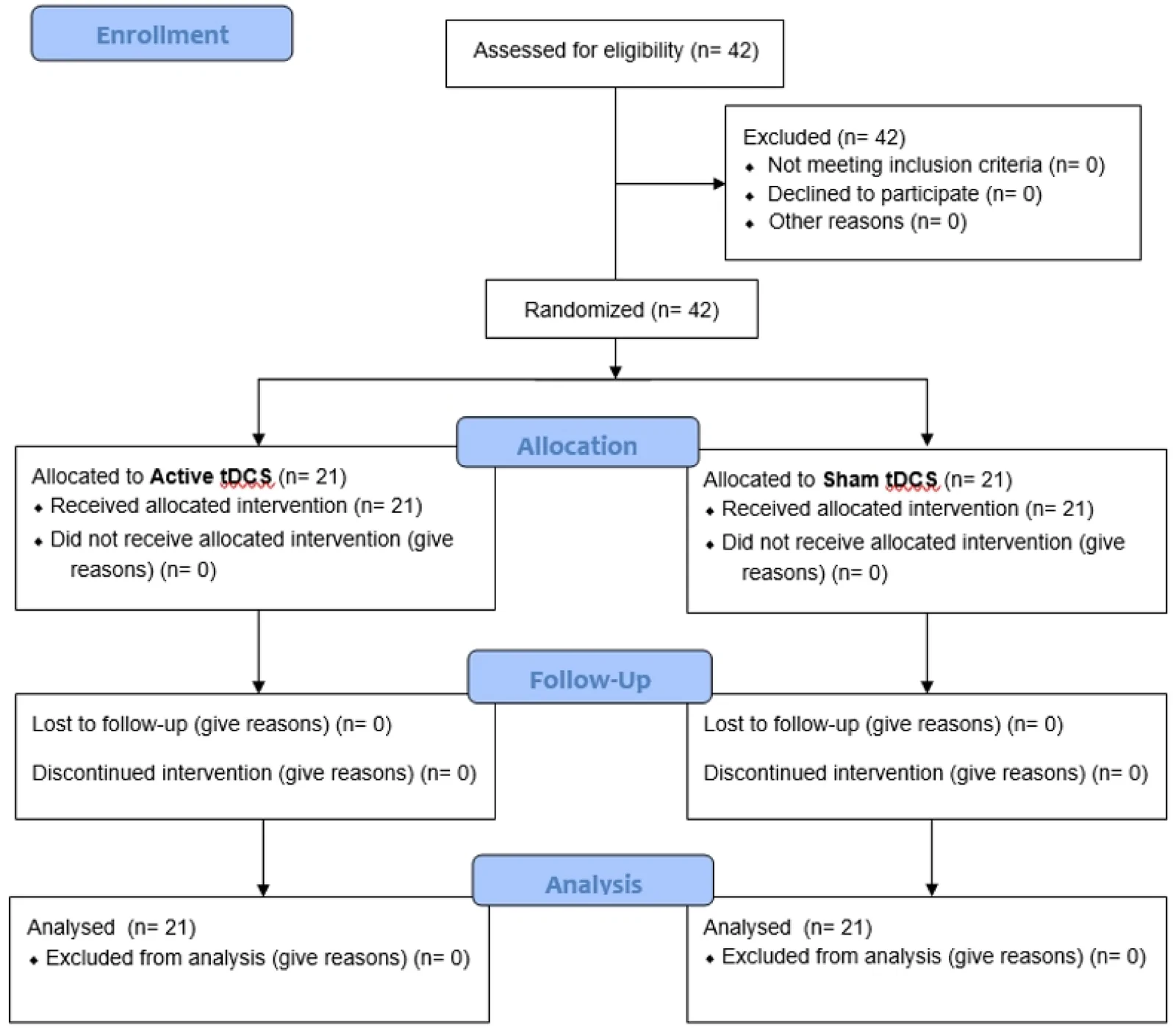
CONSORT 2010 flow diagram.

Ethical approval was granted by the Ethics Committee of the Polytechnic Institute of Santarém (approval reference: Nº3-2025ESDRM). The study was conducted in accordance with the ethical principles outlined in the Declaration of Helsinki (2013) [35]. All participants provided written informed consent prior to enrollment after being fully informed about the study objectives, procedures, potential risks, and benefits. Participants were assured of their right to withdraw from the study at any time without any consequences. Confidentiality of personal data was maintained by anonymizing all participant information and storing data securely in compliance with data protection regulations. The study adhered to the Consolidated Standards of Reporting Trials (CONSORT) guidelines to ensure transparent reporting of the randomized controlled trial [36] (Figure 1).

### 2.3. Clinical trial registration

Prior to study initiation, this RCT was registered in the UMIN Clinical Trials Registry (registration number: UMIN000057181; https://center6.umin.ac.jp/cgi-open-bin/ctr_e/ctr_view.cgi?recptno=R000065363) on March 1, 2025.

### 2.4. Sample size determination

The required sample size was calculated a priori using G*Power software (version 3.1.9.4) [23],[27], based on an expected large effect size (Cohen's d = 0.80) for between-group differences in kinematic outcomes. This effect size was selected based on previous randomized controlled trials, including Samakosh et al. (2023) and Gorji et al. (2022), which investigated biomechanical interventions for DKV and related conditions. Specifically, Samakosh et al. (2023) reported a large effect size (Cohen's d = 0.5) for improvements in knee kinematics following high-intensity training and electrical stimulation in individuals with patellofemoral pain, a condition closely associated with DKV [23]. Similarly, Gorji et al. (2022) demonstrated large effect sizes (Cohen's d = 0.80) for motor control and core stability exercises improving balance and kinematic outcomes in women with chronic low back pain, which shares biomechanical risk factors with DKV [27]. With a two-tailed alpha level of 0.05 and 80% statistical power (1−β), the analysis indicated a minimum of 21 participants per group (total N = 42). This sample size is consistent with previous randomized controlled trials investigating similar biomechanical interventions for DKV [27].

### 2.5. Randomization

Participants were randomly allocated to either the active tDCS group or the sham tDCS group using a random number generator (RNG) implemented in Excel. A simple randomization procedure was employed, where each participant was assigned a unique identifier, and the RNG generated a sequence determining group allocation (active or sham). To ensure balance between groups, the randomization sequence was checked to confirm approximately equal distribution (21 participants per group). An independent researcher, blinded to participants' baseline characteristics, performed the randomization to minimize allocation bias. Group assignments were concealed in sealed, opaque envelopes and revealed to participants only after baseline assessments were completed.

### 2.6. Procedures

The present study is a double-blind RTC, parallel-group design. To select participants, we visited the Sari Soccer Board and recruited players with at least three years of experience in the provincial league (training three times per week for 45–60 minutes) who also exhibited DKV. Following initial evaluation and DKV diagnosis using the single-leg landing (SLL) test, participants were randomized into two groups [27]. Upon confirming DKV and study entry, participants' height was measured using a wall-mounted caliper (Iran-made) and weight was recorded with a digital scale (AND 610). Performance was assessed via vertical jump and 8-hop tests (dominant leg, defined as the preferred kicking leg [37]), with a 10-minute rest between tests. A 5-minute standardized warm-up (double-leg squats: 2 sets × 8 repetitions; double-leg jumps: 2 sets × 5 repetitions; dynamic leg extensions) preceded all assessments and interventions [23]. The active tDCS group received 15 minutes of tDCS (2 mA) followed by 30 minutes of core stability exercises (3 sessions/week, 8 weeks). The sham group followed the same protocol but received 30 seconds of sham tDCS. Double-blinding was ensured for both participants and outcome assessors. Participant blinding was achieved using identical electrode setups (35 cm² saline-soaked sponges, placed at C3, C4 and FP1) for both groups, with the sham tDCS protocol delivering 30 seconds of stimulation (10-second ramp-up, 10-second hold, 10-second ramp-down) to mimic initial sensations of active tDCS. Outcome assessors, who were distinct from participants and not involved in delivering the intervention, were blinded by coding video data (for FPPA analysis) and performance results (vertical jump and 8-hop test) with participant IDs that concealed group allocation. An independent researcher managed coding and data analysis to prevent unblinding. To assess blinding efficacy, participants completed a post-intervention questionnaire asking whether they believed they received active or sham tDCS, with responses analyzed to confirm successful blinding. The criteria for study inclusion and exclusion are outlined in Table 1.

**Table 1.** Entry and exit criteria.

Entry criteria	Exit criteria
• Male gender • Age range 18 to 25 years • Having at least three years of experience in soccer having a normal body mass index (18.5 to 24.9) • Existence of dynamic valgus during SLL test • Absence of obvious abnormalities of the lower limbs (femoral anteversion, Genu valgum, Genu varum, tibial rotation and flat soles) • No history of surgery in the trunk and lower limbs • No history of permanent injury such as degenerative changes in the knee joint, chronic instability of the ankle, etc. • Absence of anterior cruciate ligament injury • Absence of lower limb injury in the past year • Failure to attend the anterior cruciate ligament injury prevention program	• Absence in two consecutive sessions or three training sessions from the entire training program • Reluctance to continue participating in exercises • Causing injury and pain during the training period or in sports activity

#### 2.6.1. Primary outcomes

The SLL task (kinematic) asked players to perform a 3 unilateral hop landing task. Once subjects were comfortable with the task, they were asked to perform 3 familiarization trials landing on the dominant leg. The hop landing task involved the subject hopping off a 30 cm high box, landing with the same leg onto a mark 30 cm from the bench and holding the position on landing for 3 s [5],[23]. A two-dimensional method was used to analyze the kinematics of the knee. The two-dimensional frontal projection plane angles of knee valgus alignment were measured [23],[29]. A digital video camera (Canon Powershot SX620HS) with video recording capability at 50 frames per second was placed at the height of the subject's knee, 3 m anterior to the subject's landing target, and aligned perpendicular to the frontal plane [17],[23]. The digital images were imported into a digitizing software program (Kinovea) and the videos were coded. The angle subtended between the lines formed between the markers at the anterior superior iliac spine and middle of the tibiofemoral joint and that formed from the markers on the middle of the tibiofemoral joint to the middle of the ankle mortise was recorded as the valgus angle of the knee. The three markers were placed on all players by the same individual. The angle was captured at the point corresponding to the lowest point of the landing descent phase [23]. The same individual digitized all the data from all subjects. The average FPPA angle value from three trials was used for analysis [23]; The rest time was 60 s between three attempts, and the rest time was 120 s between this test and the next test SLL [23].

#### 2.6.2. Secondary outcomes

*Triple Hop*. This test utilized a 6-meter-long narrow measuring tape, firmly placed on the ground. Participants performed three consecutive hops, covering the maximum possible distance, landing on the same foot each time, and holding the final landing position for at least 3 seconds. Hand movements were permitted to aid balance. After 2–3 practice attempts, participants completed two triple-hop trials with their dominant leg, and the total distance covered was recorded [37]. A 60-second rest was provided between trials, and a 120-second rest was allowed between each hop/jump test [37].

*8-Hop*. The 8-hop test was employed to assess lower extremity power, speed, and balance, focusing on single-leg control. The test course is 5 meters long and 1 meter wide, featuring seven obstacles (three at the top, three at the bottom, and one in the center) [37]. The participant starts behind the line on their dominant leg, with the nondominant leg slightly bent at the knee and hip. Upon the start signal, the participant hops through the course at maximum speed, completing two laps, and the time is recorded to the nearest 0.01 second. Participants must keep their hands on their iliac crests to prevent arm movements. The test is performed with shoes. Participants complete one to three practice attempts, followed by two scored trials with a 30-second rest between them; the best time is used for analysis. If a participant loses balance or deviates from the path, the trial is deemed invalid, and the test is repeated [37].

### 2.7. Interventions

#### 2.7.1. tDCS

**Figure 2.**
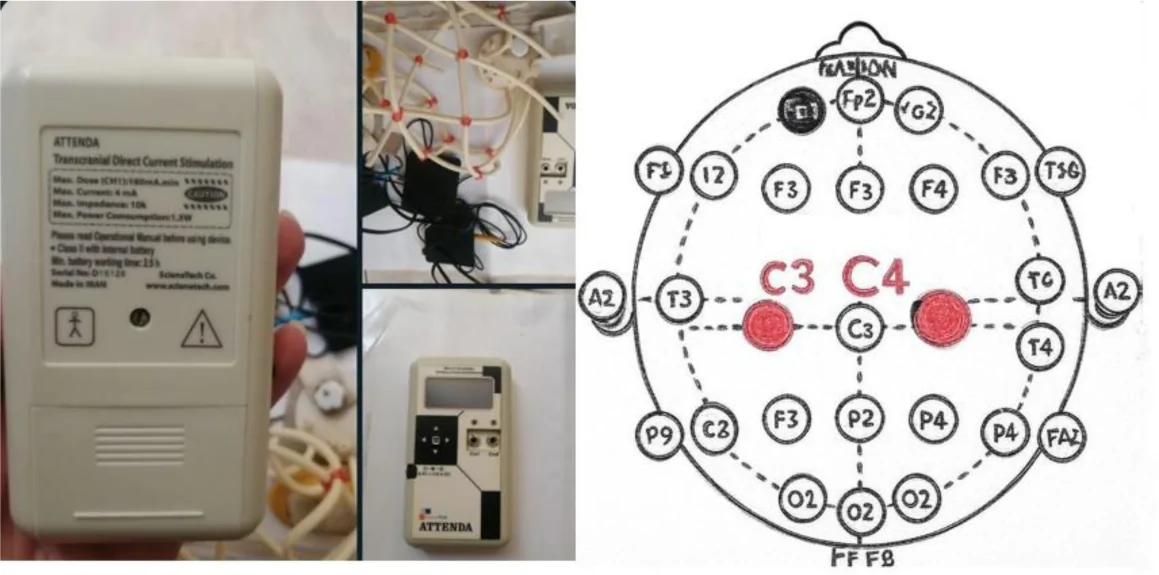
tDCS model.

The active tDCS group received 2 mA stimulation for 15 minutes per session (24 sessions) using the ATTENDA tDCS device (Iran). Electrodes (35 cm², saline-soaked sponges) were placed with the anode at C3, C4 (left and right M1) and cathode at FP1 (Figure 2). The protocol included 10-second ramp-up/down periods. The sham group received 30 seconds of stimulation (10-second ramp-up, 10-second hold, 10-second ramp-down) to mimic sensations. Blinding was assessed via a post-intervention questionnaire. Medical staff with 7 years of tDCS experience monitored sessions, with no adverse events reported [38].

#### 2.7.2. Core stability exercise

All participants, after learning about all core stability exercises, performed the exercises for 8 weeks (three sessions per week, each session 45 minutes) under the supervision of a physiotherapist. The progress in exercises was based on the principles of overload and gradual increase in the volume of exercises; Progress was made during the duration or repetition of each exercise [27]. Table 2 shows all the exercises performed over 8 weeks.

**Table 2.** Core stability exercises.

Training	(Sets and repetitions per session)							
	1–2 weeks		3–4 weeks		5–6 weeks		7–8 weeks	
	Set	R/S	Set	R/S	Set	R/S	Set	R/S
Plank (S) 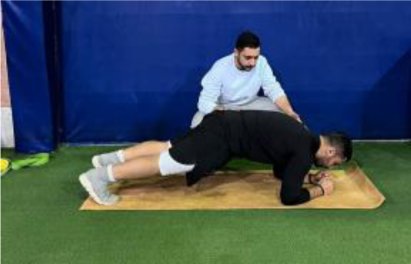	3	20	3	25	4	30	5	30
Four-legged position with raising the opposite arm and leg (R) 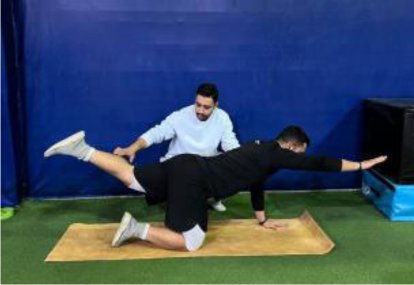	3	20	3	25	4	30	5	30
Glute bridge (R) 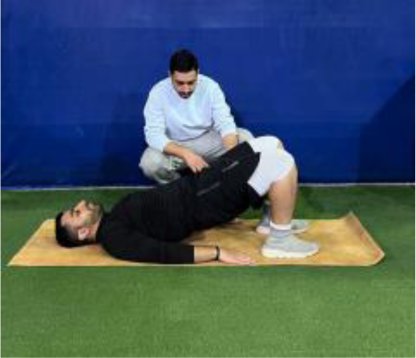	3	10	3	15	4	15	5	15
Single-leg adjusted side bridge for each side of the body (R) 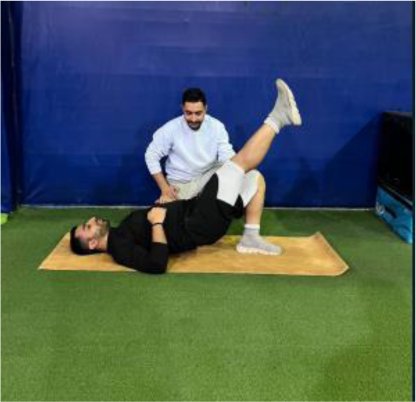	3	10	3	15	4	15	5	15
Bird dog (R) 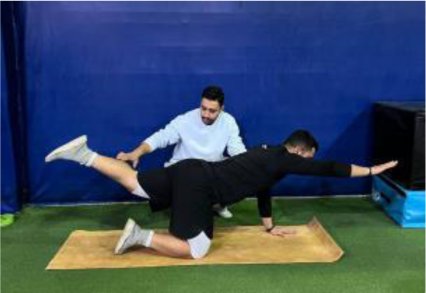	3	10	3	15	4	15	5	15
Standing on one leg with the knee flexed (S) 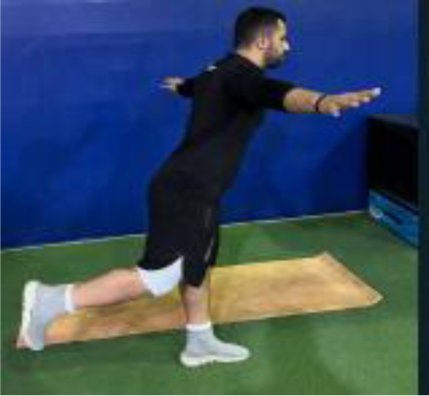	3	10	3	15	4	15	5	15
Plank with knee tap (R) 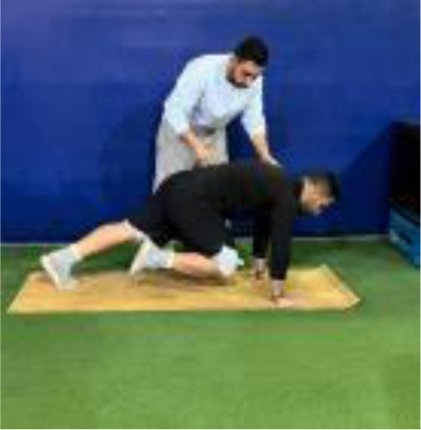	3	8	3	12	4	15	5	15
Standing with feet on the BosuBall (S) 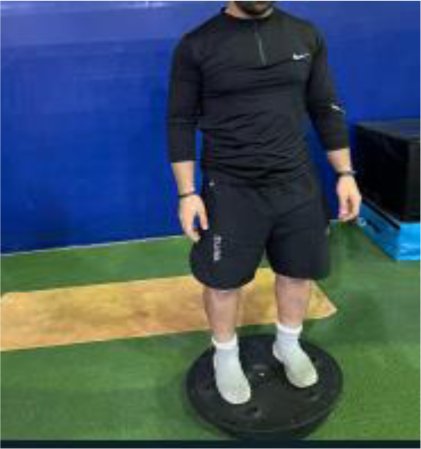	3	8	3	12	4	15	5	15
Hollow body hold (S) 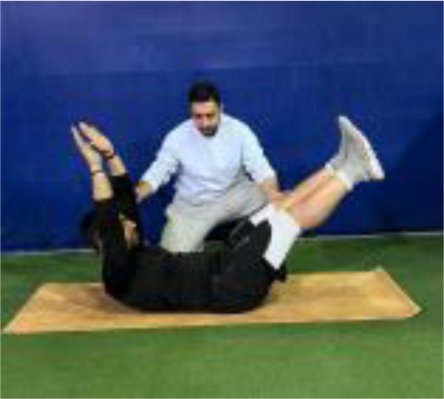	3	8	3	12	4	15	5	15
Medicine ball slam (R) 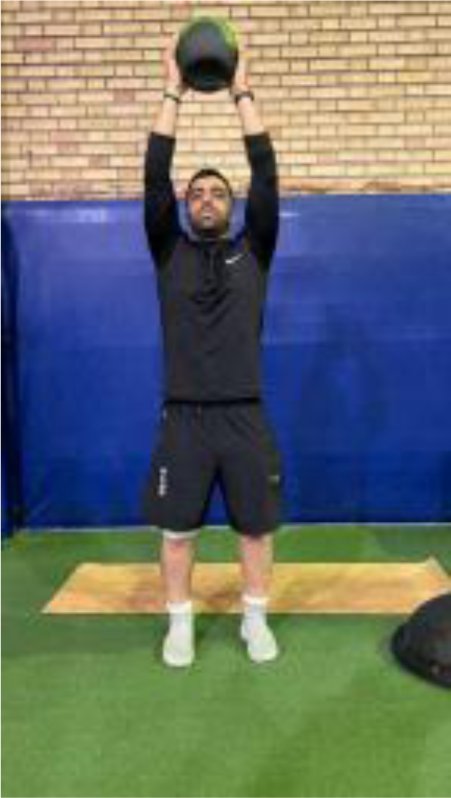	3	8	3	12	4	15	5	15
Stability ball back extension (S) 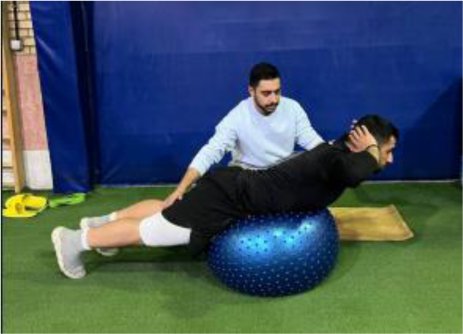	3	8	3	12	4	15	5	15

Note: S = Second, R = Repetition.

### 2.8. Statistical analysis

The Shapiro–Wilk test was used to verify the normality of the dependent variables. Levene's test was applied to assess the homogeneity of variances, confirming that the residuals of the dependent variables across groups exhibited equal variances. Scatter plots were generated to confirm a linear relationship between pre-test and post-test data for each group, satisfying the assumptions for ANOVA analysis. Following these validations, a 2 × 2 mixed-model ANOVA (treatment group × time) with Bonferroni-corrected post hoc tests were performed. For each dependent variable, results were reported as mean ± standard deviation (SD). The percentage change from baseline was calculated and compared. Statistical significance was set at a p-value < 0.05. Effect sizes were determined using Partial Eta Squared (*η*p²), with thresholds of 0.2 (small), 0.5 (moderate), and 0.8 (large) as per Cohen's guidelines [39]. Data analysis was conducted using SPSS software version 27 (IBM Corp., Armonk, NY, USA). GraphPad Prism version 8 was employed to visualize and compare pre- and post-test means between groups.

## 3. Results

Forty-two male soccer players (age: 21.8 ± 2.4 years; BMI: 23.5 ± 2.8 kg/m²) with DKV completed the study (21 per group, with no dropouts or exclusions). Baseline characteristics (age, height, weight, BMI, and years of soccer experience) were similar between groups (p > 0.05 for all comparisons, Table 3). No participants were excluded due to noncompliance or adverse events during the 8-week intervention. Recruitment occurred from March 2025 to April 2025, with follow-up completed by June 2025.

**Table 3.** Baseline participant characteristics.

Variable	Active tDCS (n = 21)	Sham (n = 21)	p-value
Age (years)	22.60 ± 2.40	21.00 ± 2.30	0.11
Height (m)	1.81 ± 0.07	1.78 ± 0.10	0.29
Weight (kg)	73.30 ± 4.90	75.80 ± 7.70	0.22
BMI (kg/m²)	22.80 ± 1.90	24.20 ± 3.40	0.13
Soccer Experience (years)	7.80 ± 2.20	7.70 ± 2.00	0.88

**Table 4.** Pre- and post-test results for primary and secondary outcomes.

Outcome	Group	Pre-test (Mean ± SD)	Post-test (Mean ± SD)	% Change	p-value	*η*p²
FPPA (degrees)	Active tDCS	166.53 ± 2.98	175.94 ± 1.91	+5.65%	<0.001	0.82
	Sham	167.37 ± 3.55	171.15 ± 3.26	+2.26%	0.012	0.25
Vertical jump (cm)	Active tDCS	21.98 ± 1.49	27.54 ± 2.57	+25.30%	<0.001	0.75
	Sham	20.95 ± 2.62	23.14 ± 3.07	+10.45%	0.008	0.22
8-hop (seconds)	Active tDCS	9.17 ± 1.01	7.24 ± 0.52	−21.05%	<0.001	0.67
	Sham	9.39 ± 0.80	8.05 ± 0.91	−14.27%	0.004	0.30

Note: *η*p²: Cohen's effect size.

**Figure 3.**
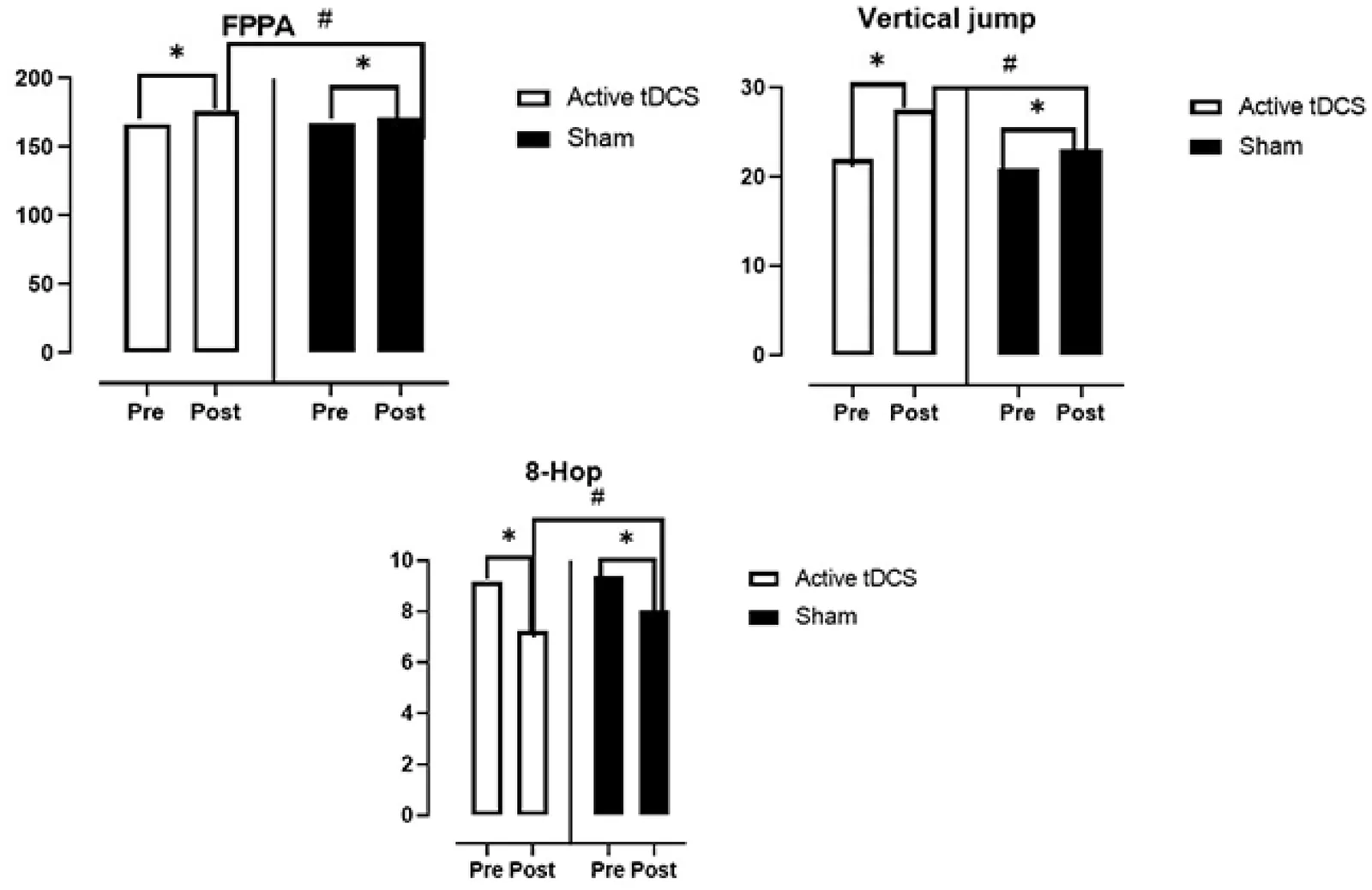
Comparison of changes over time and between groups. *There is a difference between pre-test and post-test, ^#^There is a difference between the two groups.

### 3.1. Primary outcome: Knee kinematics (SLL task)

The 2 × 2 mixed-model ANOVA revealed a significant group × time interaction for the FPPA of knee valgus alignment [F (1,40) = 8.12, p = 0.007, *η*p² = 0.17]. Post hoc tests with Bonferroni correction showed that the active tDCS group significantly increased FPPA from baseline (pre-test: 166.53 ± 2.98 degrees; post-test: 175.94 ± 1.91 degrees; p < 0.001, *η*p² = 0.82) compared to the sham group (pre-test: 167.37 ± 3.55 degrees; post-test: 171.15 ± 3.26 degrees; p = 0.012, *η*p² = 0.25). The percentage change in FPPA was +5.65% for the active tDCS group and +2.26% for the sham group (Table 3, Figure 3).

### 3.2. Secondary outcome: Vertical jump test

For the vertical jump test, a significant group × time interaction was observed for jump height [F (1,40) = 15.67, p < 0.001, *η*p² = 0.28]. Post hoc tests indicated that the active tDCS group improved significantly from baseline (pre-test: 21.98 ± 1.49 cm; post-test: 27.54 ± 2.57 cm; p < 0.001, *η*p² = 0.75) compared to the sham group (pre-test: 20.95 ± 2.62 cm; post-test: 23.14 ± 3.07 cm; p = 0.008, *η*p² = 0.22). The percentage change in jump height was +25.30% for the active tDCS group and +10.45% for the sham group (Table 4, Figure 3).

### 3.3. Secondary outcome: 8-hop test

The 8-hop test time showed a significant group × time interaction [F (1,40) = 10.45, p = 0.002, *η*p² = 0.21]. Post hoc tests revealed that the active tDCS group reduced completion time significantly (pre-test: 9.17 ± 1.01 seconds; post-test: 7.24 ± 0.52 seconds; p < 0.001, *η*p² = 0.68) compared to the sham group (pre-test: 9.39 ± 0.80 seconds; post-test: 8.05 ± 0.91 seconds; p = 0.004, *η*p² = 0.30). The percentage change in completion time was −21.05% for the active tDCS group and −14.27% for the sham group (Table 4, Figure 3).

## 4. Discussion

This study investigated the combined effects of active versus sham tDCS paired with core stability exercises on knee kinematic alignment (FPPA) and lower limb performance (vertical jump and 8-hop test) in young male soccer players with DKV. The results support our hypothesis that active tDCS, by enhancing M1 excitability, leads to greater improvements in FPPA and performance outcomes compared to sham tDCS when combined with core stability training. These findings align with emerging evidence on the role of neuromodulation and targeted exercise in improving biomechanical and functional outcomes in athletes.

### 4.1. Knee kinematics (FPPA)

The active tDCS group's greater FPPA improvement (+5.65% vs. +2.26%) suggests that tDCS enhances the effectiveness of core stability exercises in correcting DKV. Anodal tDCS over M1 increases corticospinal excitability, improving neural drive to muscles like the gluteus medius and vastus medialis, which counteract medial knee collapse [18],[40]. Specifically, tDCS enhances descending motor pathways from M1, optimizing the timing and coordination of muscle activation during dynamic tasks like landing [40]. Core stability exercises strengthen hip and trunk muscles, stabilizing the pelvis and reducing knee valgus during landing [34]. The synergy of tDCS and core exercises likely enhances proprioceptive feedback from mechanoreceptors, improving motor control and reducing DKV [18],[41]. This synergistic effect arises because tDCS primes neural circuits, increasing the responsiveness of motor neurons to subsequent core exercise-induced muscle activation, thereby amplifying biomechanical corrections. tDCS modulates descending motor pathways and enhances proprioceptive feedback, improving motor control to reduce DKV by creating a “window of opportunity” for neuroplasticity, which facilitates the consolidation of correct motor patterns learned during exercises [32]. Our results synthesize with prior literature, showing that the combined intervention outperforms standalone neuromuscular training, as reported by Moshashaei et al. (2024) (3.8% FPPA improvement, *η*p² = 0.65) and Sánchez-Barbadora et al. (2025), due to tDCS amplifying neuroplasticity and neuromuscular adaptations [18],[22]. Compared to Moshashaei et al. (2024), who reported a 3.8% improvement in knee alignment in taekwondo athletes with tDCS and neuromuscular training, our findings indicate a larger effect size (*η*p² = 0.82 vs. 0.65), likely due to the combined tDCS and core stability protocol tailored for soccer players [18]. These improvements translate to reduced ACL injury risk, as DKV is a primary noncontact injury mechanism in soccer [7]. The large effect size (*η*p² = 0.82) underscores the clinical significance of this intervention, suggesting its potential as a preventive strategy for soccer players with DKV. However, individual variability in neural plasticity or baseline DKV severity may influence response to the intervention, warranting further investigation.

### 4.2. Lower limb performance (Vertical jump and 8-hop test)

The active tDCS group showed substantial improvements in vertical jump height (+25.30% vs. +10.45% in the sham group) and 8-hop test completion time (−21.05% vs. −14.27% in the sham group), indicating enhanced lower limb power, speed, and single-leg control. These findings are consistent with recent studies highlighting tDCS's role in augmenting neuromuscular performance. For example, a 2020 study by Grandperrin et al. demonstrated that anodal tDCS over M1 increased quadriceps strength and jump performance in athletes by enhancing motor unit recruitment and reducing central fatigue [42]. Similarly, Sánchez-Barbadora et al. (2025) found that tDCS improved single-leg balance and dynamic stability in individuals with biomechanical deficits, which aligns with our 8-hop test results [22].

Core stability training likely contributed to these performance gains by improving trunk and pelvic stability, which are critical for efficient force transfer during explosive movements like jumping and hopping [43]. The combination of tDCS and core stability training may have amplified these effects by enhancing neural drive to the lower limb muscles, as evidenced by the large effect sizes (*η*p² = 0.75 for vertical jump, *η*p² = 0.68 for 8-hop test). Anodal tDCS over M1 enhances corticospinal excitability by depolarizing neuronal membranes, increasing the firing rate of motor neurons in the corticospinal tract, which improves the recruitment and synchronization of motor units in lower limb muscles, such as the quadriceps and gluteus maximus, during explosive movements like jumping and agile tasks like the 8-hop test [18],[42]. This neural facilitation reduces the perception of effort and central fatigue, enabling sustained high-intensity performance [42]. Core stability training, by strengthening muscles like the transversus abdominis, multifidus, and gluteus maximus, enhance lumbopelvic stability, optimizing force transmission from the core to the lower limbs and improving movement efficiency during dynamic tasks [34],[43]. These performance gains reflect tDCS's role in promoting neuroplasticity, enhancing motor unit recruitment, and amplifying neuromuscular adaptations from core stability training, as supported by Sánchez-Barbadora et al. (2025) and Nicolo et al. (2018) [22],[32]. The improvements have practical implications for soccer players, enhancing explosive power and dynamic stability, which may reduce injury risk during high-demand activities and improve competitive performance. The synergistic effect arises as tDCS primes the M1, increasing its responsiveness to afferent feedback from strengthened core muscles, resulting in enhanced neuromuscular coordination and power output. This synergy could be particularly beneficial for soccer players, who require rapid, coordinated movements under high physical demands.

### 4.3. Clinical and practical implications

The findings suggest that integrating active tDCS with core stability training could be a novel strategy for reducing DKV and enhancing lower limb performance in soccer players. This combined approach may offer a time-efficient intervention for amateur athletes, who often lack access to extensive rehabilitation or training programs. The significant improvements in FPPA and performance outcomes highlight the potential of this intervention to reduce ACL injury risk, given DKV's established link to noncontact ACL tears [44]. The 5.65% FPPA reduction aligns with injury risk thresholds, suggesting a clinically meaningful reduction in ACL injury risk [45]. Moreover, the absence of adverse events during the 8-week intervention supports the safety of tDCS and core stability training in this population.

Practically, implementing tDCS in sports settings requires trained personnel and specialized equipment, which may limit its accessibility. However, the portability and relatively low cost of modern tDCS devices (e.g., the ATTENDA model used in this study) make it feasible for use in sports clubs or rehabilitation centers. Future research should explore the cost-effectiveness of this intervention and its applicability to female athletes, who have a higher incidence of ACL injuries and DKV [46].

### 4.4. Limitations and future directions

While the study provides robust evidence for the efficacy of combined tDCS and core stability training, several limitations warrant consideration. First, the sample was restricted to male soccer players aged 18–25 years, limiting generalizability to female athletes or other age groups. Second, the study did not assess long-term retention of kinematic and performance improvements, which is critical for understanding the durability of the Preganancy effects. Third, while the double-blind design minimized bias, the sham tDCS protocol (30 seconds of stimulation) may not fully replicate the sensory experience of active tDCS, potentially affecting blinding efficacy. Finally, the study focused on M1 stimulation, but other brain regions (e.g., the cerebellum and prefrontal cortex) may also influence motor control and could be explored in future studies. Future research should integrate kinematic analysis with surface electromyography (EMG) to measure changes in activation patterns of hip and thigh muscles (e.g., gluteus medius, vastus medialis), confirming the hypothesized neuromuscular mechanisms. Prospective longitudinal studies are needed to determine whether these kinematic and performance improvements translate into actual reductions in ACL injury incidence over one or more competitive seasons. Additionally, studies exploring optimal tDCS parameters (e.g., intensity, duration, number of sessions) could maximize synergistic effects with core stability training, building on findings from Kidgell et al. (2013) [47]. Future research should also investigate the combined effects of tDCS with other training modalities, such as plyometric or neuromuscular training, to further enhance its efficacy in addressing DKV and related injuries.

## 5. Conclusions

This study demonstrates that active tDCS combined with core stability training significantly improves knee kinematic alignment (FPPA) and lower limb performance (vertical jump and 8-hop test) in young male soccer players with DKV, with greater benefits compared to sham tDCS. These findings highlight the potential of neuromodulation as an adjunct to exercise-based interventions for injury prevention and performance enhancement in sports. Further research is needed to confirm these results in diverse populations and to optimize the intervention for broader clinical application.

## Use of AI tools declaration

The authors declare they have not used Artificial Intelligence (AI) tools in the creation of this article.


