# Supplementary material for: Active and sham transcranial direct-current stimulation (tDCS) plus core stability on the knee kinematic and performance of the lower limb of the soccer players with dynamic knee valgus; two armed randomized clinical trial
Source: AIMS Neurosci. 2025 Jul 21;12(3):312–31. doi: 10.3934/Neuroscience.2025017 (PMC12521934; doi:10.3934/Neuroscience.2025017)
Supplement: Supplementary file 1 [file neurosci-12-03-017-s001.pdf]

Neuroscience

AIMS Neuroscience, 12(3): 312–331.
DOI: 10.3934/Neuroscience.2025017
Received: 17 May 2025
Revised: 30 June 2025
Accepted: 09 July 2025
Published: 21 July 2025

https://www.aimspress.com/journal/neuroscience

*Research article*

# Active and sham transcranial direct-current stimulation (tDCS) plus core stability on the knee kinematic and performance of the lower limb of the soccer players with dynamic knee valgus; two armed randomized clinical trial

**Hadi Mohammadi Nia Samakosh[1], Maedeh Maktoubian[2], Seyyed Pedram Rouhani Doost[3], Rafael Oliveira[4], Georgian Badicu[5,*], Sameer Badri Al-Mhanna[6], Mahdieh Hassanzadeh[7], Peyman Amadekhiar[8] and Reza Rezaeain Vaskasi[8,*]**

1 Department of Biomechanics and Corrective Exercises and Sports Injuries, University of Kharazmi, Tehran 15719–14911, Iran
2 Department of Sport Sciences and Health, Faculty of Sport Sciences and Health, University of Tehran 1417935840, Iran
3 Department of Sports Physiology, Faculty of Physical Education and Sports Sciences, University of Mazandran 1353447416, Babolsar, Iran
4 Santarém Polytechnic University, School of Sport, Rio Maior 2040–413, Portugal & Research Center in Sport Sciences, Health Sciences and Human Development (CIDESD), Santarém Polytechnic University, Rio Maior 2040–413, Portugal
5 Department of Physical Education and Special Motricity, Faculty of Physical Education and Mountain Sports, Transilvania University of Braşov, Braşov, 500068, Romania
6 Center for Global Health Research, Saveetha Medical College and Hospitals, Saveetha Institute of Medical and Technical Sciences, University of Saveetha, Chennai 602105, India
7 Department of Elementary Education, Faculty of Hazrat Fatemeh Al-Zahra, Farhangian University of Ghaemshahr 1998963341, Mazandaran, Iran
8 Department of Corrective Exercises and Sports Injuries, Faculty of Sport Sciences, University of Shomal 4616184596, Mazandaran, Iran

* **Correspondence:** Reza Rezaeain Vaskasi: Email: Rezaeianr7@gmail.com, Tel: +989360679338; Georgian Badicu: Email: georgian.badicu@unitbv.ro, Tel: +40769219271.

# Supplementary

**Table 1.** CONSORT 2010 Checklist.

| Section/Topic | Item No | Checklist item | Reported on page/section |
|---|---|---|---|
| **Title and Abstract** | 1a | Identification as a randomised trial in the title | Title: Explicitly states “two armed randomized clinical trial” (Introduction, first line). |
| | 1b | Structured summary of trial design, methods, results, and conclusions | Abstract |
| **Introduction** | 2a | Scientific background and explanation of rationale | Introduction: Describes ACL injuries, dynamic knee valgus (DKV), tDCS, and core stability training as interventions, with supporting references [1–36]. |
| | 2b | Specific objectives or hypotheses | Last paragraph Introduction |
| **Methods** | 3a | Description of trial design (such as parallel, factorial) including allocation ratio | Methods (Study Design): This study is a two-armed, double-blind, parallel-group RCT with a 1:1 allocation ratio (42 participants, 21 per group). |
| | 3b | Important changes to methods after trial commencement (such as eligibility criteria), with reasons | Not applicable |
| | 4a | Eligibility criteria for participants | Methods (Participants, Table 1) |
| | 4b | Settings and locations where the data were collected | Methods (Procedures): Recruitment from Sari Soccer Board, with assessments conducted at a sports facility in Sari, Iran. |
| | 5 | The interventions for each group with sufficient details to allow replication, including how and when they were actually administered | Methods (Interventions): Describes active tDCS (2 mA, 15 min, anode at $C_{3,4}$, cathode at FP1) and sham tDCS (30 s ramp-up/down), followed by core stability training (30 min, 3x/week, 8 weeks). Table 2 details exercise progression. |
| | 6a | Completely defined pre-specified primary and secondary outcome measures, including how and when they were assessed | Methods (Primary and Secondary Outcomes): Primary outcome: FPPA during single-leg landing (SLL) task, assessed via 2D video analysis at baseline and post-intervention. Secondary outcomes: Vertical jump height and 8-hop test time, with detailed assessment protocols. |
| | 6b | Any changes to trial outcomes after the trial commenced, with reasons | Not applicable |

| | | |
|---|---|---|
| 7a | How sample size was determined | Methods (Sample Size Determination): Calculated using G*Power, expecting a large effect size (Cohen's d = 0.80), α = 0.05, power = 80%, resulting in 21 participants per group (N = 42). |
| 7b | When applicable, explanation of any interim analyses and stopping guidelines | Not applicable |
| 8a | Method used to generate the random allocation sequence | Methods (Randomization): Random Number Generator (RNG) implemented in Excel. |
| 8b | Type of randomisation; details of any restriction (such as blocking and block size) | Methods (Randomization): Simple randomization was used without restrictions. |
| 9 | Mechanism used to implement the random allocation sequence (such as sequentially numbered containers), describing any steps taken to conceal the sequence until interventions were assigned | Methods (Randomization): Allocation was concealed using sealed, opaque envelopes prepared by an independent researcher, opened only after baseline assessments. |
| 10 | Who generated the random allocation sequence, who enrolled participants, and who assigned participants to interventions | Methods (Randomization): An independent statistician generated the allocation sequence, an orthopedic specialist enrolled participants, and a physiotherapist assigned interventions. |
| 11a | If done, who was blinded after assignment to interventions (for example, participants, care providers, those assessing outcomes) and how | Methods (Study Design): Double-blind trial, with participants and researchers unaware of group assignments. Outcome assessors were blinded by coding video data and anonymizing participant identities during analysis. |
| 11b | If relevant, description of the similarity of interventions | Methods (Interventions): Sham tDCS mimicked real tDCS with 30 s ramp-up/down to ensure similar tactile sensations, ensuring blinding. |
| 12a | Statistical methods used to compare groups for primary and secondary outcomes | Methods (Statistical Analysis): Describes 2×2 mixed-model ANOVA (group × time) with Bonferroni-corrected post hoc tests, reporting mean ± SD, percentage change, and partial eta squared ($\eta p^2$). |
| 12b | Methods for additional | Not applicable |

| | | | |
|---|---|---|---|
| | | analyses, such as subgroup analyses and adjusted analyses | |
| **Results** | 13a | For each group, the numbers of participants who were randomly assigned, received intended treatment, and were analysed for the primary outcome | Results: 42 participants randomized (21 per group), all received intended treatment and were analyzed for primary outcome (no exclusions). |
| | 13b | For each group, losses and exclusions after randomisation, together with reasons | Results: No losses or exclusions reported. |
| | 14a | Dates defining the periods of recruitment and follow-up | Results |
| | 14b | Why the trial ended or was stopped | Not applicable; trial completed as planned. |
| | 15 | A table showing baseline demographic and clinical characteristics for each group | Results (Table 3): Reports baseline characteristics (age, height, weight, BMI, soccer experience) with p-values showing no significant differences. |
| | 16 | For each group, number of participants (denominator) included in each analysis and whether the analysis was by original assigned groups | Results: All 42 participants (21 per group) included in analyses, conducted per original group assignments (intention-to-treat). |
| | 17a | For each primary and secondary outcome, results for each group, and the estimated effect size and its precision (such as 95% confidence interval) | Results (Table 4, Chart 1): Reports pre- and post-test means ± SD, percentage change, p-values, and $\eta p^2$ for FPPA, vertical jump, and 8-hop test. 95% CIs for effect sizes added in Table 4. |
| | 17b | For binary outcomes, presentation of both absolute and relative effect sizes | Not applicable |
| | 18 | Results of any other analyses performed, including subgroup analyses and adjusted analyses, distinguishing | Not applicable |

| | | | |
|---|---|---|---|
| | | pre-specified from exploratory | |
| | 19 | All important harms or unintended effects in each group | Results: No adverse events reported; monitored by medical staff during tDCS and exercise sessions. |
| **Discussion** | 20 | Trial limitations, addressing sources of potential bias, imprecision, and, if relevant, multiplicity of analyses | Discussion (Limitations and Future Directions) |
| | 21 | Generalisability (external validity, applicability) of the trial findings | Discussion (Limitations and Future Directions) |
| | 22 | Interpretation consistent with results, balancing benefits and harms, and considering other relevant evidence | Discussion (Clinical and Practical Implications) |
| **Other Information** | 23 | Registration number and name of trial registry | Methods (Code of Ethics): UMIN Clinical Trials Registry, UMIN000057181, registered March 1, 2025. |
| | 24 | Where the full trial protocol can be accessed, if available | Methods (Clinical Trial Registration): Available at UMIN Clinical Trials Registry (https://center6.umin.ac.jp/cgi-open-bin/ctr_e/ctr_view.cgi?recptno=R000065363). |
| | 25 | Sources of funding and other support (such as supply of drugs), role of funders | This study was not funded by any institution |

AIMS Press

© 2025 the Author(s), licensee AIMS Press. This is an open access article distributed under the terms of the Creative Commons Attribution License (https://creativecommons.org/licenses/by/4.0)
